# Network Anatomy Controlling Abrupt-like Percolation Transition

**DOI:** 10.1038/s41598-017-00242-4

**Published:** 2017-03-13

**Authors:** Hirokazu Kawamoto, Hideki Takayasu, Misako Takayasu

**Affiliations:** 1 Department of Computational Intelligence and Systems Science, Interdisciplinary Graduate School of Science and Engineering, Tokyo Institute of Technology 4259, Nagatsuta-cho, Yokohama 226-8502 Japan; 2 Institute of Innovative Research, Tokyo Institute of Technology 4259, Nagatsuta-cho, Yokohama 226-8502 Japan; 3 Sony Computer Science Laboratories, 3-14-13, Higashi-Gotanda, Shinagawa-ku, Tokyo 141-0022 Japan

## Abstract

We virtually dissect complex networks in order to understand their internal structure, just as doctors do with the bodies of animals. Our novel method classifies network links into four categories: bone, fat, cartilage, and muscle, based on network connectivity. We derive an efficient percolation strategy from this new viewpoint of network anatomy, which enables abrupt-like percolation transition through removal of a small amount of cartilage links, which play a crucial role in network connectivity. Furthermore, we find nontrivial scaling laws in the relationships between four types of links in each cluster and evaluate power exponents, which characterize network structures as seen in the real large-scale network of trading business firms and in the Erdős-Rényi network. Finally, we observe changes in the transition point for random bond percolation process, demonstrating that the addition of muscle links enhances network robustness, while fat links are irrelevant. These findings aid in controlling the percolation transition for an arbitrary network.

## Introduction

Different networks, such as human and business relationship networks, and power networks, are everywhere in our world^1–4^. Most complex networks in social systems can be categorized as having scale-free and small-world properties^5, 6^. Many methods quantifying such inhomogeneous networks have been proposed from various fields including biology, information science and physics^4, 6–9^. It is important to understand how a network can be made robust under attack, including methods for intentional removal of nodes and links because such networks form the basis of the society and economy^10–14^. Thus, it is necessary to determine what elements contribute to reinforcing network connectivity, and to find practical ways to enhance robustness of the system.

Percolation theory has been studied in the fields of mathematics and physics to clarify macroscopic connectivity from a microscopic viewpoint^4, 15–20^. Specifically, the percolation transition properties of complex networks have been attracting the attention of many scientists since the proposal of the scale-free network model^5^. It has been reported that a scale-free network is fragile against targeted attacks, but robust against random failures^10^. Furthermore, recent percolation models have been extended to explain a discontinuous percolation transition (DPT)^2, 18, 19, 21, 22^. It has been suggested that a real power network carries the risk of massive blackouts due to cascading failures in a multi-layered network^2^, and discontinuity in the explosive percolation model has attracted great interest in recent years due to its simple yet diverse characteristics^23–25^.

Classification of nodes and links, such as community extraction, is also an important field of study, and comprehensive graphical expressions have become available for this application^26–30^. In the theory of percolation transition for square lattices, there are studies classifying “backbone links” based on significant contribution to overall connectivity^31–33^, however, such classifications have not been yet introduced to percolation study in complex networks.

In this study, we further generalize the anatomical concept of a “backbone” by introducing a novel “network anatomy”, which virtually dissects any given complex network and classifies its links based on their contribution to network connectivity. In the Method section, all network links are classified into four categories: bone, fat, cartilage, and muscle links, as an analogy for the anatomy of animal bodies. In the Result 1 section, we show that a percolation strategy assembled from these link categories enables the abrupt-like transition in a large-scale real network, as well as artificial networks. Nontrivial scaling laws are observed among the four classified link types and scaling exponents that characterize a network are shown in the Result 2 section. In the Result 3 section, we observe shifts of the percolation transition point caused by doping fat and muscle links to clarify the functional roles of each link type. Finally, we discuss the abruptness and controllability of a percolation transition by utilizing our link classifications, and summarize our findings.

### Method: Classification of links based on network robustness

**Figure 1:**
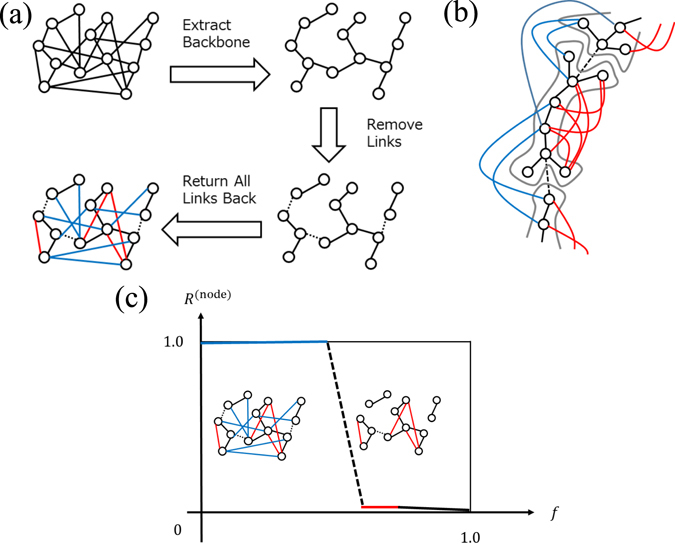
(**a**) Schematic figure of link classification process. For a given network, a spanning tree is computed; then the network is decomposed into many clusters by removing links. Finally, all links are returned to their original location. (**b**) Schematic figure of clusters. The bone links (black lines) form the skeleton of each cluster. The fat links (red lines) connect two nodes within a cluster. The cartilage links (black dotted lines) connect clusters to an element of a backbone. The muscle links (blue lines) are links that connect two different bone clusters after the final procedure of returning links to their original positions. (**c**) Schematic figure showing the relationship between the order parameter *R* ^(node)^, and the control parameter for the anatomical percolation. Firstly, muscle links are removed randomly (blue line). Secondly, cartilage links are removed randomly (black dotted line). Thirdly, fat links are removed randomly (red line). Finally bone links are removed randomly (black line). The order parameter mainly changes during the second process of removing cartilage links.

Here we explain our method for categorizing links. The entire process is divided into three steps. First, extraction of a backbone. Second, decomposition into clusters. Third, returning all removed links to the network as shown in Fig. 1(a), which is applicable to arbitrary networks, including directed and unconnected networks. Initially, we construct a spanning tree, which is a tree-like subnetwork connecting all nodes without loops, and classify it as a backbone. We introduce three popular methods for constructing the spanning tree: Breadth First Search Tree (BFST), Random Cost minimum Spanning Tree (RCST) and Depth First Search Tree (DFST). In BFST, we start with the node that has the largest degree and create links between nodes by following a breadth first search^34^. In RCST, we assign each link a uniform random number as a cost and compute a spanning tree that minimizes the total cost of the network using Prim’s algorithm^35^. In DFST, we select the node that has the largest degree and create links between nodes by following a depth first search^36^.

Next, we remove links from the spanning tree one by one, and decompose the network into separated clusters. Links are removed in a near-random fashion: however, we attempt not to remove links that will create an isolated node with no links as much as possible. We repeat this procedure until the normalized largest cluster size becomes smaller than the threshold size, which is 0.01 times the original size. And also the size of the second largest cluster becomes the largest by choosing a link in the current largest cluster as a candidate for link removal. The links remaining after this procedure are called “bone links”, as they are elements of the backbone, and form the skeleton of each cluster. Links removed in this process are called “cartilage links”, as they are elements of the spanning tree, and connect clusters of bone links.

Finally, we return all the removed links to the clusters of bone links. The links connecting two nodes within a cluster of bone links are called “fat links”, and increase redundancy within each cluster, which does not contribute to global connectivity. Returned links that connect two different clusters of bone links are called “muscle links”, and increase redundancy in the whole network. Following this procedure, all links are classified as bone, fat, cartilage, or muscle links. For each cluster, bone links form the skeleton of the cluster and fat links play the role of bypasses within the cluster (Fig. 1(b)). The links connecting different clusters of bone links are either cartilage or muscle links, and this difference is derived from the definition of the backbone.

It should be noted that this classification is not unique based on the choice of the spanning tree and the removed links. In supplementary information S1, we show that there are links which are automatically classified as bones, and also there are links which are highly likely to be classified as muscles. By performing 100 trials, it can be shown that this classification is correlated with the shell-decomposition, which quantitatively characterizes nodes based on number of links and mutual connectivity^4, 37, 38^. Links in the lower shell tend to be classified as bones or cartilages, while those in the higher shell are more likely to belong to be classified as muscles. In addition, we calculate the distribution of the entropy for all links, and confirm that links are consistently categorized to some extent.

### Result 1: Abrupt-like Change of Largest Cluster Size in Novel Percolation Strategies

We propose new percolation strategies that can enable abrupt-like changes in order parameters based on the link classifications introduced in the previous section. We remove links to define a percolation process following the order of muscle, cartilage, fat and bone links. In the first stage, links are chosen from the set of muscle links at random and removed one-by-one. After all the muscle links are removed, cartilage links are removed in the same way, followed by fat links, and finally bone links. We call this an anatomical percolation. As a basic order parameter, we adopt the parameter *R* ^(node)^ which is defined as the number of nodes in the largest cluster divided by the total number of nodes *N*. We use the ratio of the number of removed links divided by the total number of links *M* as a control parameter.

In the first stage of removing muscle links, cartilage links maintain overall network connectivity and the largest cluster size is the same as the initial network size even when all muscle links have been removed (Fig. 1(c)). In the second stage of removing cartilage links, it is expected that the network will be rapidly broken into many separate clusters, and the largest cluster size will change abruptly. Finally, the network becomes a set of isolated nodes through the removal of fat and bone links. It should be noted that cluster size, defined by number of contained nodes, does not change during the fat link removal step, because these links only connect nodes within each cluster.

**Figure 2:**
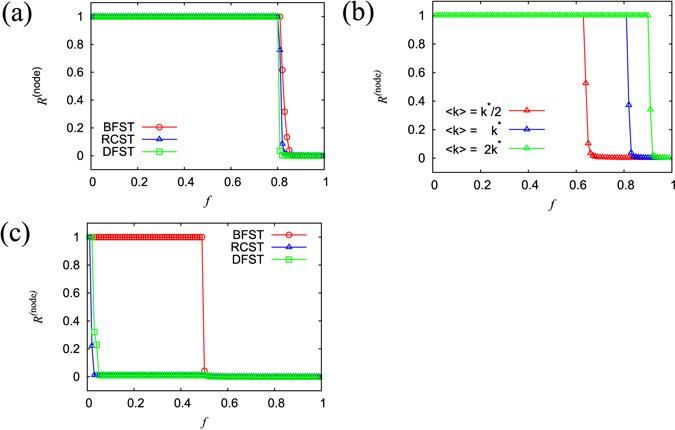
(**a**) Change in the largest cluster size *R* ^(node)^ in a business relations network from Japan. BFST (red circle), RCST (blue triangles) and DFST (green squares). Each plot represents the average of 100 trials. (**b**) Change in the largest cluster size *R* ^(node)^ in an Erdős-Rényi network. The average degree of connectivity 〈*k*〉 is represented by k*/2 (red triangles), k* (blue triangles), and 2k* (green triangles), where k* is the average degree for the business relations network. Each plot represents the average of 10 trials. (**c**) Change in the largest cluster size *R* ^(node)^ in an extended ring network. BFST (red circle), RCST (blue triangles) and DFST (green squares). Each plot represents the average of 10 trials.

Now we apply this anatomical percolation strategy to a real large-scale network. Here, we use a Japanese business relations network from 2009, which consists of 446,360 nodes (firms) and 2,388,582 links (transactions). The average degree of connectivity 〈*k*〉 = k* is calculated as 10.7. This data has been provided by TEIKOKU DATABANK, Ltd., a Japanese credit research company. This network is considered a typical complex network with both scale-free and small-world properties^3^. This network is made up of 15.6% bone links, 0.370% fat links, 3.10% cartilage links and 80.9% muscle links when we adopt RCST as a backbone. We achieved similar results when using other types of spanning trees (BFST, DFST). Using the proposed percolation process, we find an abrupt-like change in the order parameter *R* ^(node)^ and the value of *R* ^(node)^ suddenly approaches near zero around *f* $\simeq$ 0.81, where the process of removing muscle links finishes as shown in Fig. 2(a). These changes can be more sharply observed in the ordering of BFST, RCST and DFST. This is because the ratio of cartilage links to total links is smaller using this ordering. As shown in Supplementary Information S2, this transition is similar to the conventional percolation transition for this network as well as a configuration model network, in the sense that cluster size distributions follow power law distributions at the transition point. These results suggest that our transition is the continuous percolation transition exhibiting critical behaviors.

We perform the simulation on other networks to confirm universality of the abrupt-like percolation transition observed in the business relations network. We apply anatomical percolation to Erdős-Rényi network^39^ where each link is connected with a given probability. We prepare three kinds of networks where the number of links for each is 110,000, and network density is k*/2, k*, and 2k*, respectively. Here, k* is 10.7, the average degree of connectivity for the previously used business relations network, and we adopt RCST as a backbone. As shown in Fig. 2(b), we confirm the occurrence of sharp changes for these three networks, we confirm that the ratio of muscle links to total links increases, and we confirm that the abrupt-like transition point elevates as network density increases. It is found that the network breaks down rapidly around *f* $\simeq$ 0.81, which is nearly the same as in the real network. This result suggests that the abrupt-like transition point, representing the sum of muscle and cartilage links divided by total links, does not depend on detailed network structure.

Next, we perform the simulation on an extended one-dimensional ring network (*N* = 50,000, *M* = 100,000) where each node connects to its four nearest neighbors. In this case, we find that there is a large difference in abrupt-like transition points depending on the type of the backbone used (Fig. 2(c)). While the abrupt-like transition occurs at a small *f* (less than 0.05) in both RCST and DFST, it occurs around *f* $\simeq$ 0.50 in BFST. This is because allocation of surplus links, which are not elements of the backbone, differs based the spanning tree method used. In RCST and DFST surplus links are classified as fat links, while in BFST they are classified as muscle links.

We show that the ratio of each type of link to total links does not depend on the method used for computing the spanning tree of a real network, but that it does depend on the method used for computing the spanning tree in an extended ring network. This result suggests that the method for computing a backbone does have the ability to control the abrupt-like transition point.

**Figure 3:**
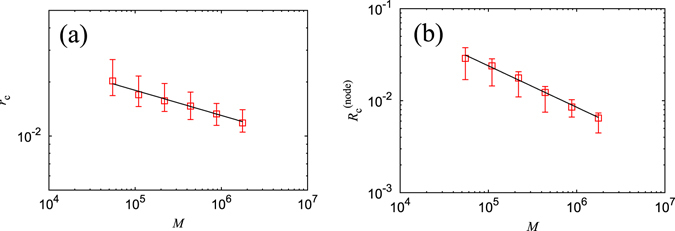
(**a**) The system size *M* vs, the ratio *r*_c_ of the number of cartilage links to the total number of links in a log-log plot. The solid line shows the power exponents of 0.14. (**b**) The system size *M* vs, the normalized largest cluster size *R*_*c*_^(node)^ immediately after removing all of the cartilage links in a log-log plot. The solid line shows the power exponents of 0.45.

Finally, we consider the finite size dependency of the anatomical percolation transition to estimate scaling relations for the sharpness of the order parameter *R* ^(node)^ around the transition point. Because it is easy to change the system size for Erdős-Rényi networks, we use this type of networks for the finite-size analysis. We prepare six networks that each have the same average degree of connectivity with different system sizes. Here, we introduce threshold conditions that cause the largest cluster size to become less than 4 × *N* ^−0.5^ during the process of cartilage link classification by removing links in the spanning tree network. We calculate the ratio *r*_*c*_ of the number of the cartilage links to total links and the normalized largest cluster size *R*_*c*_^(node)^after all cartilage links have been removed. These quantities correspond to the horizontal width of the black dotted line, and the height of the red line respectively in Fig. 1(c). In this simulation, we observe scaling laws for both quantities, *r*_*c*_ ∝ *M* ^−α^ and *R*_*c*_^(node)^ ∝ *M* ^−*β*^ as shown in Fig. 3(a,b). The exponents *α* and *β* are estimated to be 0.14 and 0.45 respectively. This result shows that both quantities vanish at the limit of an infinite system size. Specifically, at the infinite limit the order parameter changes sharply from 1 to 0. We here call this sharp change the abrupt-like percolation transition.

### Result 2: Scaling Laws among the four Types of Links

In this section, we show novel scaling laws observed among the four link types. First, we denote the numbers of bone, fat, cartilage and muscle links as *m*_b_, *m*_f_, *m*_c_ and *m*_m_ respectively for each cluster. Note that cartilage and muscle links are counted twice by different clusters whose nodes are connected by these link types. Here, we discuss the following scaling laws with exponents *a*_f_, *a*_c_ and *a*_m_.

1 $${m}_{{\rm{f}}}\propto {m}_{{\rm{b}}}^{{a}_{{\rm{f}}}}$$

2 $${m}_{{\rm{c}}}\propto {m}_{{\rm{b}}}^{{a}_{{\rm{c}}}}$$

3 $${m}_{{\rm{m}}}\propto {m}_{{\rm{b}}}^{{a}_{{\rm{m}}}}$$

**Figure 4:**
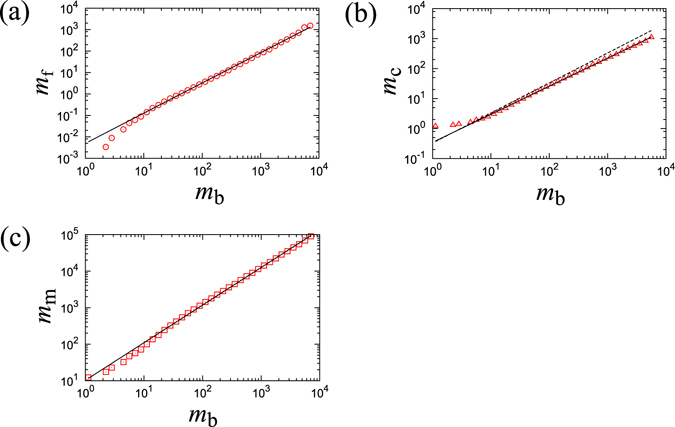
Scaling relationships for the real business network. (**a**) The number of bone links *m*_b_ vs, the number of fat links *m*_f_ by cluster in log-log scale. Plots are averaged using log scaled bins over 100 trials. The guideline shows the power law with a scaling exponent of 1.4. Error bars are omitted because the interquartile range (IQR) is as large as the size of the symbols in most plots. (**b**) The number of bone links *m*_b_ vs, the number of cartilage links *m*_c_ by cluster in log-log scale. Plots are averaged using log scaled bins over 100 trials. The guideline shows a power law with the scaling exponent of 0.94. Error bars are omitted for the same reason as in the previous plot. The dotted line shows a slope of 1.0 for reference. (**c**) The number of bone links *m*_b_ vs, the number of muscle links *m*_m_ by cluster in log-log scale. Plots are averaged using log scaled bins over 100 trials. The guideline shows the power law with a scaling exponent of 1.0. Once more, error bars have been intentionally omitted.

We observed the above three relations in the Japanese business relations network using a backbone computed by RCST. Scaling laws can be confirmed for all three relations for nearly three decades. The power exponent *a*_f_ in the relationship shown by Eq. (1), between the number of bone links *m*_b_ and fat links *m*_f_ is estimated as 1.4 (Fig. 4(a)). Here, we apply the minimum least squares method for logarithmically transformed values. This result indicates that the number of fat links that form loops in each cluster nonlinearly increases with the size of the cluster. The power exponent *a*_c_ in the relationship between the number of bone links *m*_b_ and cartilage links *m*_c_ is estimated as 0.94 (Fig. 4(b)), which is less than 1.0. Because we obtain ${m}_{{\rm{c}}}/{m}_{{\rm{b}}}\propto {m}_{{\rm{b}}}^{{a}_{{\rm{c}}}-1}$ from Eq. (2) the relative ratio of cartilage links becomes negligible as the system size increases within the condition *a*_c_ < 1. The power exponent *a*_m_, in the relation between the number of bone links *m*_b_ and muscle links *m*_m_, is estimated as 1.0 (Fig. 4(c)), and we find that the number of muscle links linearly increases with the cluster size. Therefore, muscle links exist at a constant rate independent of the cluster size.

**Figure 5:**
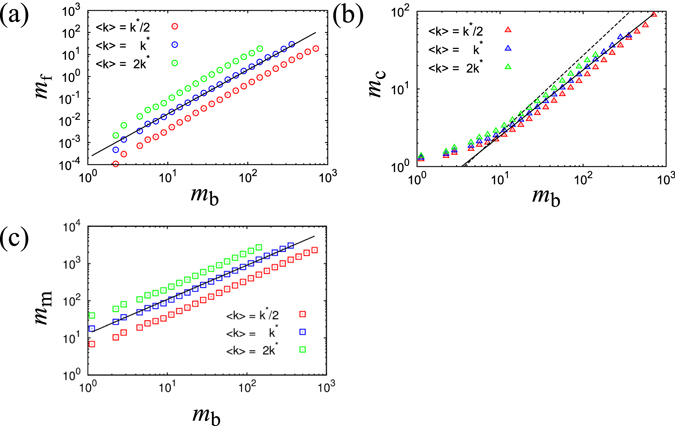
Scaling relationships for Erdős-Rényi networks. (**a**) The number of bone links *m*_b_ vs, the number of fat links *m*_f_ by cluster in log-log scale. Plots are averaged using log scaled bins over 1,000 trials. The guideline shows the power law with a scaling exponent of 2.0. (**b**) The number of bone links *m*_b_ vs, the number of cartilage links *m*_c_ by cluster in log-log scale. Plots are averaged using log scaled bins over 1,000 trials. The guideline shows the power law with a scaling exponent of 0.85. The dotted line shows a slope of 1.0 for reference. (**c**) The number of bone links *m*_b_ vs, the number of muscle links *m*_m_ by cluster in log-log scale. Plots are averaged using log scaled bins over 1,000 trials. The guideline shows the power law with a scaling exponent of 0.92.

Next, we calculate the above relationships for three variants of Erdős-Rényi networks, which we already analyzed in the previous section. Similar scaling exponents are observed for all three relationships, and we find that the change in average degree of connectivity simply shifts the transition point. This suggests that scaling exponents do not depend on probability of link connection *p* = *M*/*N*(*N*−1) between two nodes. The scaling exponent *a*_f_ in Eq. (1) is estimated as 2.0 (Fig. 5(a)), which is the same as that for a complete graph. The scaling exponent *a*_c_ in Eq. (2) is estimated as 0.85 (Fig. 5(b)), which is lower than 1.0, just like the result for the real network. The scaling exponent *a*_m_ in Eq. (3) is estimated as 0.92 (Fig. 5(c)), which is also the same as the real network. These scaling exponents are considered to have the same values for the different parameters of Erdős-Rényi networks.

In the Supplementary Information S3 we show scaling relationships for two more artificial networks, an extended one-dimensional ring network and a configuration network with a power law degree distribution. We summarize the ratios of bone, fat, cartilage and muscle links, and as well as the scaling exponents for all cases.

### Result 3: Enhancing Robustness by Doping Links

**Figure 6:**
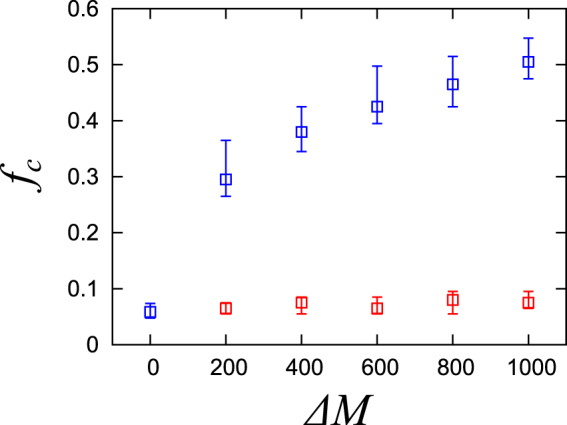
The number of doping links Δ*M* vs, the median for the percolation transition point *f*_c_ in the extended ring network. Red represents doped fat links and blue represents doped muscle links. The error bars indicate the interquartile range (IQR) for 100 trials.

Based on the extended ring network (*N* = 50,000, *M* = 100,000), we consider doping links in order to reveal the roles of categorized links for network robustness. We calculate changes in the finite-size transition point for the random bond percolation process when we dope fat or muscle links for a network whose links have already been classified as bone, fat, cartilage, and muscle links. Here, we consider bone and cartilage links to be known, and we add muscle and fat links to the network. In the initial state (Δ*M* = 0), where the links are not doped, the transition point is small (${{f}_{{\rm{c}}}}^{(rand)}\simeq 0.06$) and the system is considered fragile. However, by doping muscle links, the transition point increases and robustness is enhanced (Fig. 6). This is because doped muscle links generate global loops that connect different bones and reinforce network connectivity. It should be noted that the transition point increases to *f*_*c*_^(*rand*)^ ≃ 0.5 by doping only 1.0% links of the initial links (Δ*M* = 1,000). This example shows that doping a small amount of muscle links efficiently enhances network robustness. The transition point does not change, however, when we dope fat links in the system. This should make sense intuitively because fat links connect nodes within the same cluster of bone links and those links do not enhance global network connectivity. As a result, we find that muscle links play a crucial role in strengthening network robustness. Fat links, however, only reinforce connectivity within each cluster, but they do not contribute to global network robustness.

## Discussion

We observed the sharpness of an order parameter for abrupt-like percolation transition in the Result 1 section. Here, we discuss the properties of this transition by using the scaling exponents shown in the Result 2 section. For the scaling relation ${m}_{{\rm{c}}}/{m}_{{\rm{b}}}\propto {m}_{{\rm{b}}}^{{a}_{{\rm{c}}}-1}$, derived in Eq. (2) the number of cartilage links is somewhat negligible for networks such as *a*_c_ < 1 when considering large network size. This means that the width of the sharp change, which is equal to the ratio of cartilage links to total links becomes negligible under this condition. Therefore, the transition observed in the Result 1 section becomes a sharpness limit percolation transition at the infinite scale limit because the exponent *a*_c_ is estimated to be less than 1.0 in both the business relations network and Erdős-Rényi networks. Conversely, the percolation transition may not be sharp in a system with *a*_c_ ≥ 1. Whether or not this transition will be sharp is characterized by the exponent *a*_c_.

The transition point in this anatomical percolation is expected to be lower than that of an ordinary random percolation^4^. This is because our link classification enables the avoidance of links that do not contribute to global connectivity, so we can define a more efficient percolation transition. This process can be considered a weighted percolation process, such as a targeted attack removing nodes in descending order by degree of connectivity. Continued research may reveal an even more efficient percolation procedure that could further lower the transition point.

Finally, we discuss the controllability of this abrupt percolation transition. We found that the distribution ratio for links depends on network density. Specifically, we confirmed that the ratio of muscle links increases and the transition point elevates as the average degree of connectivity increases in Erdős-Rényi network as shown in the Result 1 section. We also demonstrated that the distribution ratio for links depends on the method chosen for computing a backbone in the example of an extended one-dimensional ring network, shown in the Result 1 section. The transition point is shown to be tunable when using the new percolation strategy even with a fixed distribution ratio for links. The strategy of removing all cartilage links after all muscle links have been removed exhibited the lowest transition point *f*_c_ with the sharpest change. Furthermore, the transition point can be controlled artificially by changing the removal order for links. For example, we are able to control the transition point freely within the range of *f*_c_ ≤ *f* ≤ *f*_c_^(*rand*)^. Here, *f*_c_^(*rand*)^ denotes the transition point on random bond percolation process. Additionally, we can tune a transition property by altering density through removal of cartilage links: for example, we can change the sharp transition shown in the Result 1 section into the slower transition of an ordinary random percolation^4^. Following the methodology discussed above, we are able to manage the percolation process of any given complex network once all links have classified as the four types defined in this new system of “network anatomy.” The analysis given in this paper provides a new framework for bridging sharp and slow transitions from the wider viewpoint of abrupt-like percolation.

## Conclusion

In this study, we introduced a general classification for links in complex networks into four types based on the viewpoint of network robustness, and defined by the percolation process. These four types of links have a functional role for network connectivity. The bone links form the skeleton of each cluster. The cartilage links maintain network connectivity in small numbers, and play a central role for the abrupt-like percolation transition. The fat links generate local redundancy and have little influence on overall network robustness. The muscle links generate global redundancy and enhance overall network robustness. Thus, our proposed model provides an intuitive understanding of network robustness through a familiar in topological structure.

The network anatomy introduced in this paper reveals interesting physically properties. In the Result 2 section, we discovered novel scaling laws, and estimated scaling exponents. We discussed the conditions that cause the transition to become sharp at the large scale limits by using the scaling exponents. It was also confirmed that doping new muscle links efficiently enhances network robustness.

## Electronic supplementary material

Supplementaly information
