# Supplementary material for: Network Anatomy Controlling Abrupt-like Percolation Transition
Source: Sci Rep. 2017 Mar 13;7:163. doi: 10.1038/s41598-017-00242-4 (PMC5428047; doi:10.1038/s41598-017-00242-4)
Supplement: Supplementary file 1 — Supplementaly information [file 41598_2017_242_MOESM1_ESM.pdf]

Supplementary Information

Network Anatomy Controlling Abrupt-like Percolation Transition

Hirokazu Kawamoto[1], Hideki Takayasu[2, 3], and Misako Takayasu[1, 2]

[1]Department of Computational Intelligence and Systems Science, Interdisciplinary Graduate School of Science and Engineering, Tokyo Institute of Technology 4259, Nagatsuta-cho, Yokohama 226-8502, Japan
[2]Institute of Innovative Research, Tokyo Institute of Technology 4259, Nagatsuta-cho, Yokohama 226-8502, Japan
[3]Sony Computer Science Laboratories, 3-14-13, Higashi-Gotanda, Shinagawa-ku, Tokyo 141-0022, Japan

S1. Basic properties of anatomical link classifications and their relationship with shell decomposition

The classifications introduced in the Method section are not uniquely determined, but depend on the method chosen for generating a spanning tree and removing links. Basically, we choose links randomly during the removal process, so the results depend on random numbers, meaning that we can determine the probability of a given link belonging to a particular category. For the Japanese business relations network, we repeated our calculations over 100 times with RCST using different random numbers and observed the probability of links being classified as bone, cartilage, fat, or muscle.

In Fig. S1, we plot the probabilities for 48 example links. In Fig. S1 (a), we plot the probabilities for eight links that are likely to be classified as bones, and in Fig. S1 (f) we plot the eight links that are likely to be classified as muscles. These links are chosen by applying K-shell decomposition in the following manner. For a positive integer K, the K-shell is defined by a set of nodes that belong to the k-core, but not to the $(k+1)$-core. The k-core is defined as the largest subgraph having a minimal degree of connectivity k [1, 2]. It is known that the Japanese business relations network can be decomposed into 25 shells [3]. We choose links that are connecting a pair of nodes within the K-shell. Figure. S1 shows the cases of (a) $K = 1$, (b) $K = 2$, (c) $K = 4$, (d) $K = 6$, (e) $K = 15$, and (f) $K = 25$. For the special case of $K = 1$, the links automatically belong to the spanning tree, so the ratios for fat and muscle are 0. If a node only has a single link, then the link becomes a bone with ratio 1.

To confirm the relationship between the anatomical classification and the shell decomposition, we plot the distribution of probabilities for each classification. Fig. S2 (a) shows distribution of probabilities to be classified as bone for all links (red), for the links in the $K = 2$ shell (blue) and for the links in the $K = 4$ shell (green). Probability distributions for the other classifications: fat, cartilage, and muscle, are shown in Fig. S2 (b), (c), and (d) respectively. The probability for a link to be classified as fat is generally low as shown in plot (d). Links are also more likely to be classified as muscle in larger shells.

Finally, we calculate the entropy to investigate the consistency of this categorization. The entropy H_{ij} of a link between node i and j is defined as follows.

$$H_{ij} = -\sum_{T} p_{ij}(T) \log_2 p_{ij}(T) \tag{1}$$

Here, T denotes link type (bone, fat, cartilage and muscle), and $p_{ij}(T)$ shows the probability to be classified as each link type in given trials. This entropy measurement is equal to 0 if a link is categorized into a particular link type in every trial. On the other hand, this measurement is equal to $\log_2 4(= 2)$ if a link is evenly categorized into the four link types. We calculate this entropy for all links, and show the distribution in Fig. S3. As a result, the average entropy is obtained as 0.56, and the distribution is located far from $\log_2 4$. Therefore, we conclude that our method is effective to classify links with certain consistency. Additionally, we calculate these distributions for each shell, and we confirm that the entropy decreases with larger shell numbers.

S2. Cluster size distributions near the transition points

To demonstrate the similarities between our transition and a conventional percolation transition, we show the distributions of cluster sizes c. This is defined as the number of connecting nodes at the point, $f_{\mathrm{ab}} = (M_{\mathrm{m}} + M_{\mathrm{c}})/M$ where M_{m} is the number of muscle links and M_{c} is the number of cartilage links. In Fig. S4 (a) and (b), the cumulative cluster size distributions are plotted in log-log scale for the Japanese business relations network and three configuration model networks, showing that they can be approximated by power laws with an exponent close to 1.5. This is the same exponent value as that of the mean field value for an ordinary percolation transition. It should be noted that the cumulative distribution for degree of connectivity for the configuration networks: $(N, M) = (95, 291, 114, 813), (192, 034, 232, 450)$, and $(384, 293, 466, 270)$, follow a power law whose exponent is 1.5. This is very close to that of the Japanese business relations network. In Fig. S4 (c), the cumulative cluster size distribution is plotted in log-log scale for the one-dimensional ring network following an exponential function. This result is consistent with the result for the one-dimensional lattice [4].

S3. Scaling laws for an extended one-dimensional ring network and a configuration network

We calculate the scaling laws for the four types of links in an extended one-dimensional ring network and a configuration network.

In the case of the ring network, we prepared a network of size $N = 50,000, M = 100,000$ and performed 100 trials. In Fig. S5 (a) non-trivial scaling relationships are found for fat links and depend on the method used for computing a backbone, in the case of BFST (red circle), the estimated exponent is 3.8. In the case of RCST (blue circles), the exponent is estimated to be 1.0. In Fig. S5 (b), the relationships between cartilage links and bone links are plotted for BFST (red circle) and RCST (blue circles). The plots are not on a straight line; therefore, we cannot define power exponents because the estimated power exponents are less than 0.1. For muscle links, we can confirm power law relationships for both BFST (red circle) and RCST (blue circles) as shown in Fig. S5 (c) with the exponents 0.97 and 0.11, respectively.

In the case of the configuration network that we used in S2, the average degree of connectivity $\langle k \rangle$ is calculated as 2.4, which is relatively small. This network has a very small number of fat links, and no scaling relationship can be found for those links. For cartilage and muscle links, we can confirm scaling relationships: $m_{\mathrm{c}} \propto m_{\mathrm{b}}^{a_{\mathrm{c}}}$ and $m_{\mathrm{m}} \propto m_{\mathrm{b}}^{a_{\mathrm{m}}}$. As shown in Fig. S6 (a), (b), the exponents a_{c} and a_{m} are both estimated to be 1.0.

Finally, we list the ratios of each link type in the following tables, with the estimated scaling exponents defined in Equations (1) - (3). For the Japanese business relations network, the ratio of muscle links remains nearly the same regardless of the method chosen for constructing spanning trees as shown in Table S1. The scaling law for m_{b} and m_{c} cannot be observed over a wide range for DFST. Table S2 shows that the ratio of muscle links increases with an increasing average degree of connectivity, which means that the transition points in the anatomical percolation process also increase. As shown in Table S3, the distribution of ratios for each link type depends on method chosen for constructing spanning trees in the extended one-dimensional ring network. However, the results for both RCST and DFST are very similar. As shown in Table S4, the

configuration model network is characterized by many cartilage links, which means that the ratio for that link type is relatively large when compared to other networks.

Table S1: The percentage of bone, fat, cartilage, and muscle links and the exponents defined in Equations (1) - (3) for the Japanese business relations network. "-" indicates that the scaling law is not observed over a wide range.

	bone	fat (a_f)	cartilage (a_c)	muscle (a_m)
BFST	13.8	0.193 (1.4)	4.88 (1.2)	81.1 (0.98)
RCST	15.6	0.370 (1.4)	3.10 (0.94)	80.9 (1.0)
DFST	18.3	0.527 (1.0)	0.36 (-)	80.8 (0.86)

Table S2: The percentage of bone, fat, cartilage, and muscle links and the exponents defined in Equations (1) - (3) for use of RCST on the Erdős-Rényi Networks.

	bone	fat (a_f)	cartilage (a_c)	muscle (a_m)
$k^*/2$	31.6	0.0623 (2.0)	4.65 (0.86)	63.7 (0.94)
k^*	15.3	0.0839 (2.0)	2.89 (0.85)	81.7 (0.92)
$2k^*$	7.32	0.0997 (2.0)	1.77 (0.85)	90.8 (0.90)

Table S3: The percentage of bone, fat, cartilage, and muscle links and the exponents defined in Equations (1) - (3) for the extended one-dimensional ring networks.

	bone	fat (a_f)	cartilage (a_c)	muscle (a_m)
BFST	49.3	0.153 (3.8)	0.734 (< 0.1)	49.8 (0.97)
RCST	49.2	48.0 (1.0)	0.754 (< 0.1)	2.04 (0.11)
DFST	49.2	46.6 (1.1)	0.825 (0.13)	3.36 (0.18)

Table S4: The percentage of bone, fat, cartilage, and muscle links and the exponents defined in Equations (1) - (3) for use of RCST on the configuration model network. "†" shows that we cannot determine the scaling law due to the low number of fat links.

	bone	fat (a_f)	cartilage (a_c)	muscle (a_m)
$N = 95,291$	63.9	$< 10^{-2}$ (†)	19.1 (1.0)	17.0 (1.0)
$N = 192,034$	65.2	$< 10^{-2}$ (†)	17.5 (0.99)	17.4 (1.0)
$N = 384,293$	65.6	$< 10^{-2}$ (†)	16.8 (0.99)	17.6 (1.0)

References

[1] Seidman, S. B. Network structure and minimum degree. *Social Networks* **5**, 269-287 (1983).

[2] Kitsak, M. *et al.* Identification of influential spreaders in complex networks. *Nat. Phys.* **6**, 888-893 (2010).

[3] Kawamoto, H., Takayasu, H., Jensen, H. J. & Takayasu, M. Precise Calculation of a Bond Percolation Transition and Survival Rates of Nodes in a Complex Network. *PLoS ONE* **10(4)**, e0119979; DOI:10.1371/journal.pone.0119979 (2015).

[4] Stauffer, D. & Aharony, A. *Introduction to Percolation Theory* (Taylor & Francis, London, 1994).

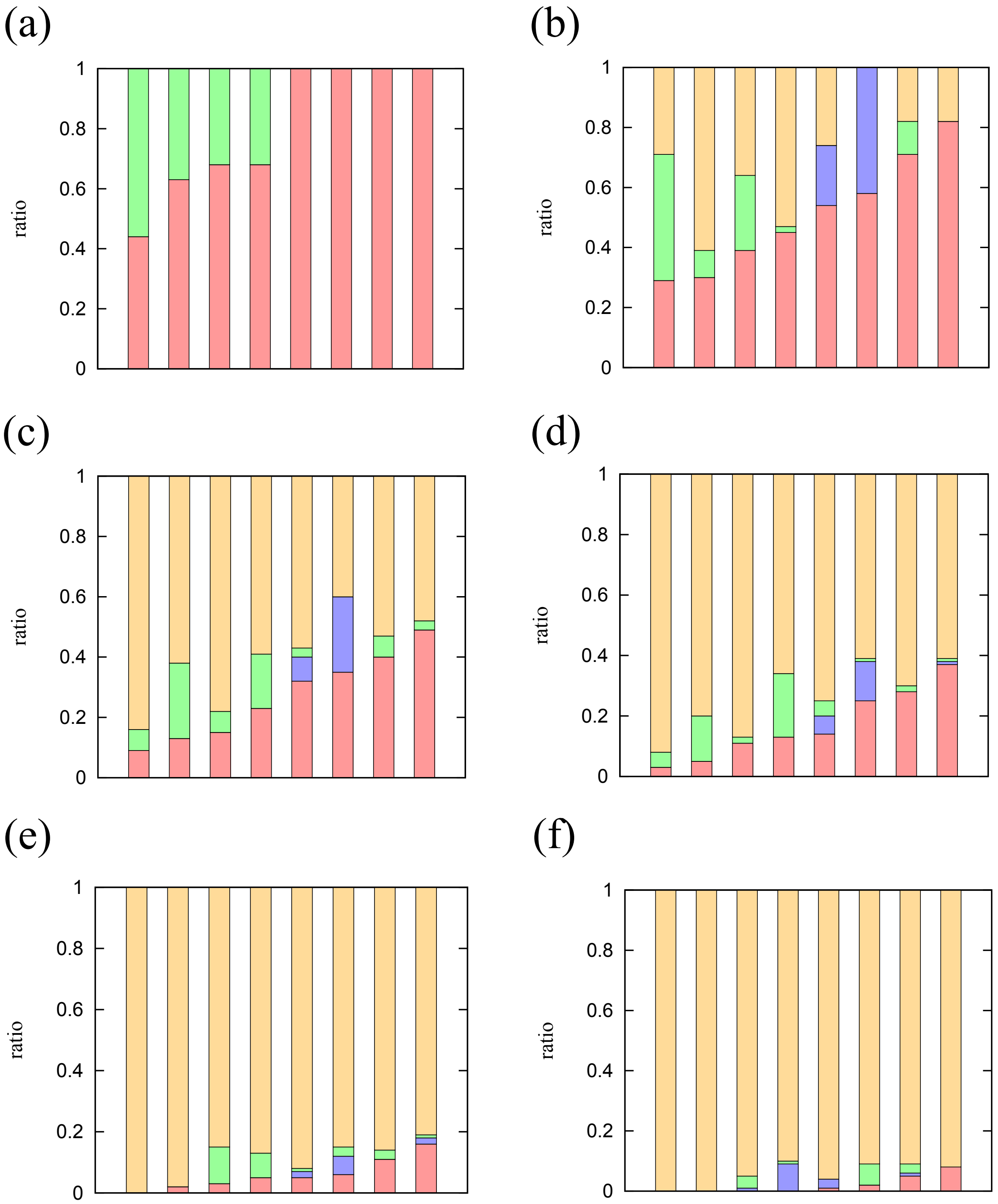

Figure S1: The probability of being each classification type for eight extracted links. The red, blue, green and orange colors represent the bone, fat, cartilage and muscle links respectively. (a) The links that have $K = 1$. (b) The links that have $K = 2$. (c) The links that have $K = 4$. (d) The links that have $K = 6$. (e) The links that have $K = 15$. (f) The links that have $K = 25$.

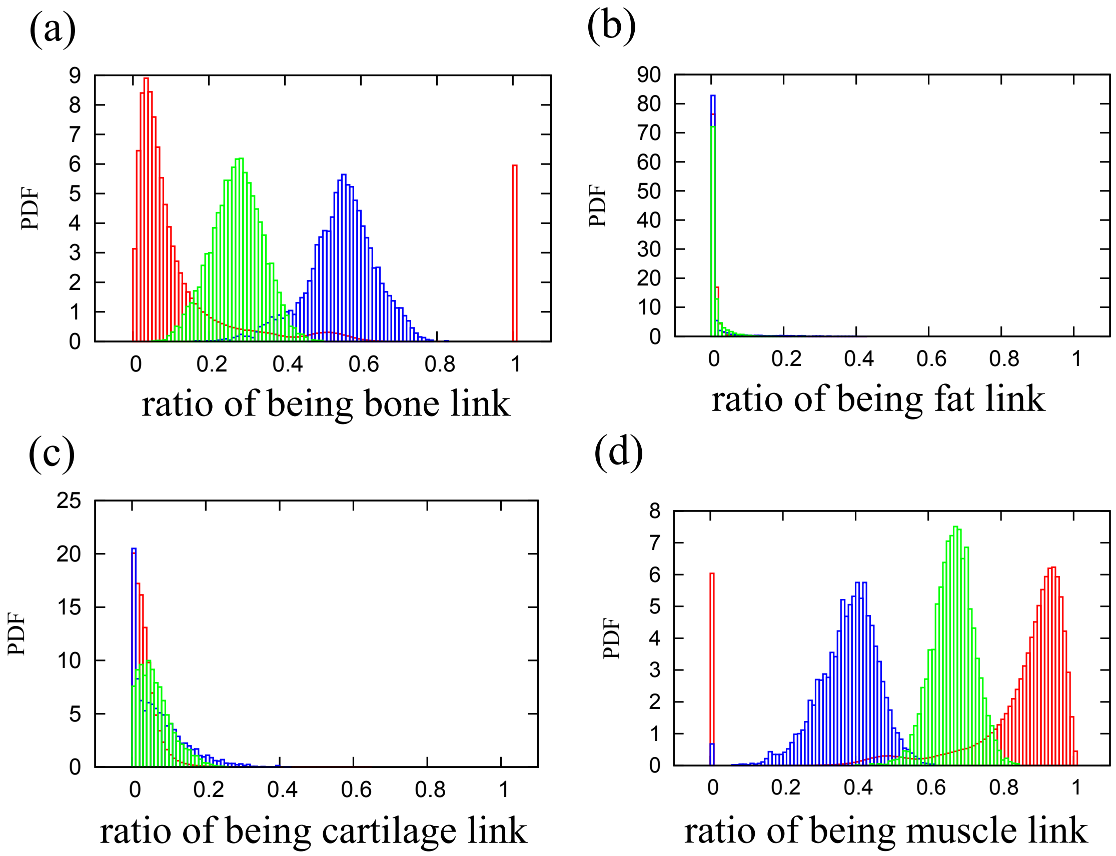

Figure S2: The probability distribution functions (PDF) for each link type. The red, blue and green colors represent all links, the links in the $K = 2$ shell, and the links in the $K = 4$ shell respectively. (a) Bone links. (b) Fat links. (c) Cartilage links. (d) Muscle links.

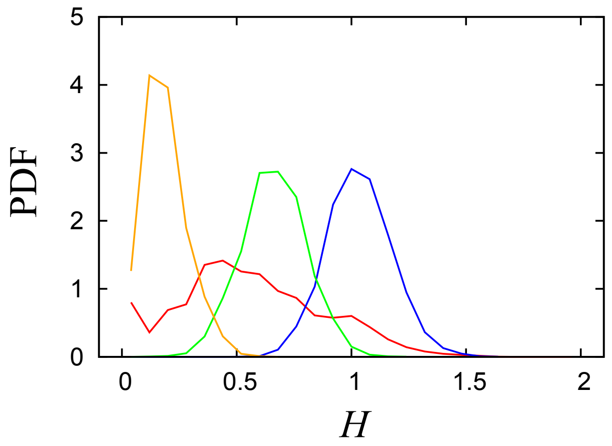

Figure S3: The probability distribution functions (PDF) of the entropy. The red, blue, green and orange colors represent all links, the links in the $K = 5$ shell, the links in the $K = 10$ shell, and the links in the $K = 25$ shell, respectively.

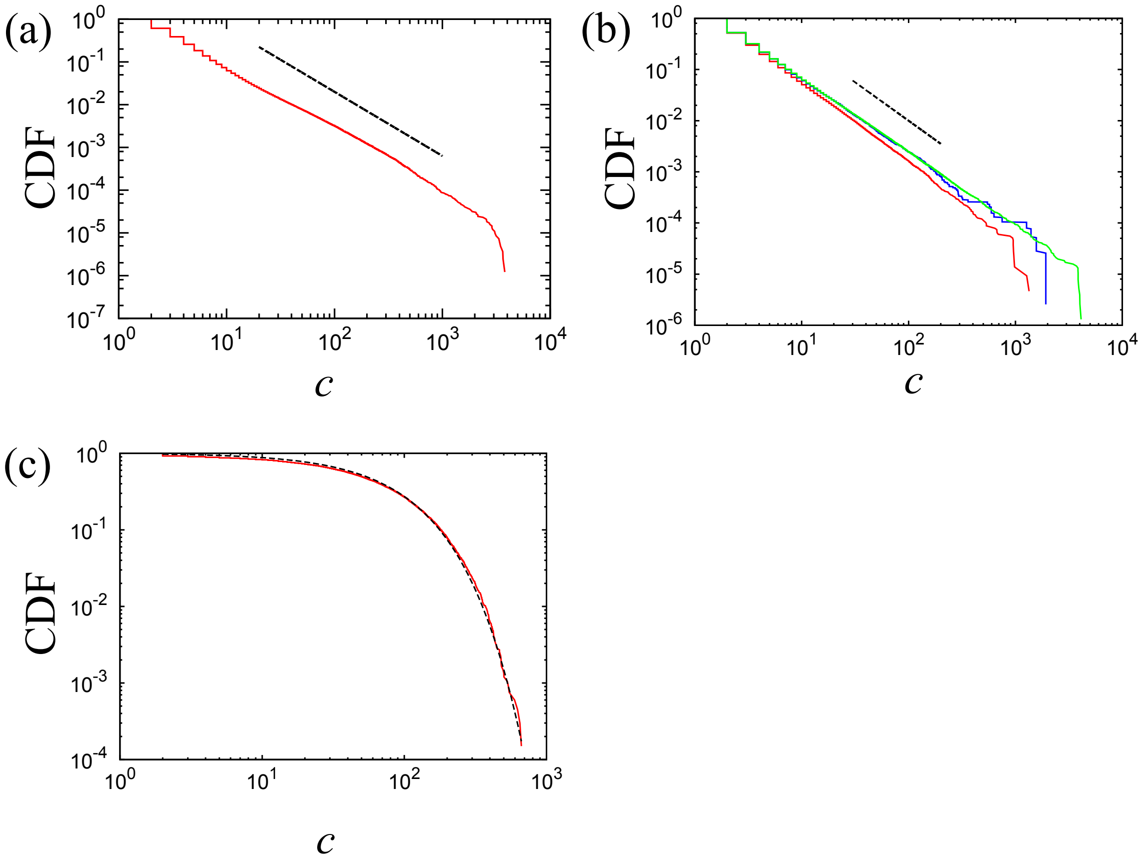

Figure S4: Cumulative cluster size distributions near the transition points. The results displayed are a superposition of 10 different trials. (a) The Japanese business relations network. In log-log scale, the guideline shows a power law with a slope of the theoretical value, 1.5. (b) The configuration model network. $N = 95,291$ (red), $N = 192,034$ (blue), and $N = 384,293$ (green). In log-log scale, the guideline shows a power law with a slope of the theoretical value, 1.5. (c) The extended one-dimensional ring network. In log-log scale, the guideline shows an exponential distribution proportional to $\exp(0.013c)$.

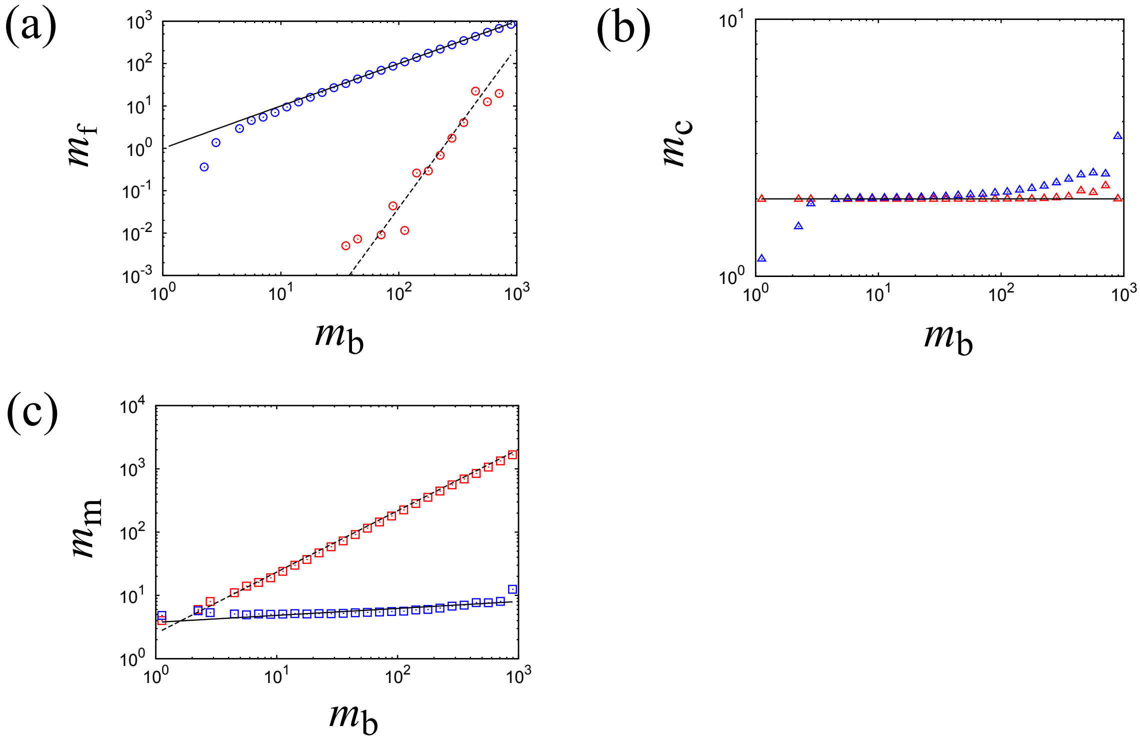

Figure S5: Scaling relationships for an extended one-dimensional ring network. (a) The number of bone links m_b vs, the number of fat links m_f by cluster in log-log scale. BFST (red circles), and RCST (blue circles). Plots are averaged using log scaled bins over 100 trials. The solid and dotted guidelines show the power law with the scaling exponent of 1.0 and 3.8 respectively. (b) The number of bone links m_b vs, the number of cartilage links m_c by cluster in log-log scale. BFST (red triangles), and RCST (blue triangles). Plots are averaged using log scaled bins over 100 trials. The solid line shows a constant value. (c) The number of bone links m_b vs, the number of muscle links m_m by cluster in log-log scale. BFST (red squares), and RCST (blue squares). Plots are averaged using log scaled bins over 100 trials. The solid and dotted guidelines shows the power law with a scaling exponent of 0.11 and 0.97 respectively.

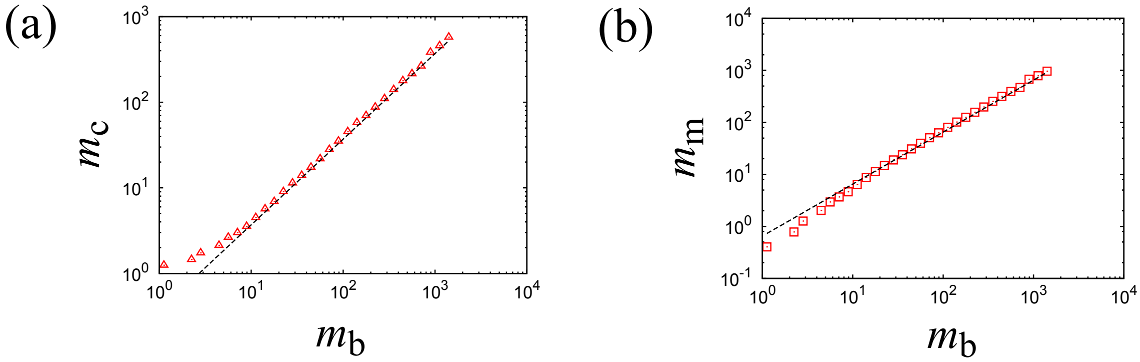

Figure S6: Scaling relationships for a configuration network, $(N, M) = (95, 291, 114, 813)$. (a) The number of bone links m_b vs the number of cartilage links m_c by cluster in log-log scale. Plots are averaged using log scaled bins over 100 trials. The guideline shows the power law with a scaling exponent of 1.0. (b) The number of bone links m_b vs the number of muscle links m_m by cluster in log-log scale. Plots are averaged using log scaled bins over 100 trials. The guideline shows the power law with a scaling exponent of 1.0.
